# Assessment of the Relationship between the Shape of the Lateral Meniscus and the Risk of Extrusion Based on MRI Examination of the Knee Joint

**DOI:** 10.1371/journal.pone.0159156

**Published:** 2016-07-14

**Authors:** Arkadiusz Szarmach, Piotr Luczkiewicz, Monika Skotarczak, Mariusz Kaszubowski, Pawel J. Winklewski, Jaroslaw Dzierzanowski, Maciej Piskunowicz, Edyta Szurowska, Bogusław Baczkowski

**Affiliations:** 1 2-nd Department of Radiology, Medical University of Gdansk, Gdansk, Poland; 2 2-nd Clinic of Orthopaedics and Kinetic Organ Traumatology, Medical University of Gdansk, Gdansk, Poland; 3 Department of Economic Sciences, Faculty of Management and Economics, Gdansk University of Technology, Gdansk, Poland; 4 Institute of Human Physiology, Medical University of Gdansk, Gdansk, Poland; 5 Department of Neurosurgery, Medical University of Gdansk, Gdansk, Poland; 6 1-st Department of Radiology, Medical University of Gdansk, Gdansk, Poland; Harvard Medical School/BIDMC, UNITED STATES

## Abstract

**Background:**

Meniscus extrusion is a serious and relatively frequent clinical problem. For this reason the role of different risk factors for this pathology is still the subject of debate. The goal of this study was to verify the results of previous theoretical work, based on the mathematical models, regarding a relationship between the cross-section shape of the meniscus and the risk of its extrusion.

**Materials and Methods:**

Knee MRI examination was performed in 77 subjects (43 men and 34 women), mean age 34.99 years (range: 18–49 years), complaining of knee pain. Patients with osteoarthritic changes (grade 3 and 4 to Kellgren classification), varus or valgus deformity and past injuries of the knee were excluded from the study. A 3-Tesla MR device was used to study the relationship between the shape of the lateral meniscus (using slope angle, meniscus-cartilage height and meniscus-bone angle) and the risk of extrusion.

**Results:**

Analysis revealed that with values of slope angle and meniscus-bone angle increasing by one degree, the risk of meniscus extrusion raises by 1.157 and 1.078 respectively. Also, an increase in meniscus-cartilage height by 1 mm significantly elevates the risk of extrusion. At the same time it was demonstrated that for meniscus-bone angle values over 42 degrees and slope angle over 37 degrees the risk of extrusion increases significantly.

**Conclusions:**

This was the first study to demonstrate a tight correlation between slope angle, meniscus-bone angle and meniscus-cartilage height values in the assessment of the risk of lateral meniscus extrusion. Insertion of the above parameters to the radiological assessment of the knee joint allows identification of patients characterized by an elevated risk of development of this pathology.

## Introduction

The menisci are fibrocartilaginous disks that play an important role in force distribution across the knee. In knees with osteoarthritis, the menisci are often ruptured or even completely macerated which might progress to their external shift (extrusion)[1–4]. The disruption of the meniscus causes the inability to generate hoop stress during load transmission and reduces mechanical protection of the cartilage. For this reason, meniscal extrusion is a risk factor for osteoarthritis progression and knee symptoms [3–6]. Although meniscal tears are often not associated with symptoms, meniscal extrusion is strongly associated with frequency of knee pain because it may mechanically stress the joint capsule with pain receptors [4,7]. Although there is a strong association between degenerative radial meniscal tears and extrusion it has been recently demonstrated that meniscal tear is the only predisposing factor to extrusion [8,9]. Other factors reported to contribute to the external shift of the meniscus are a varus alignment of the lower limb [10], joint space narrowing [5,11,12] and tibial and meniscal morphology [13]. Clinical studies are often not sufficient to establish predictors of meniscal extrusion because their cross-sectional and retrospective nature leaving the causality analysis in the sphere of speculation [3,14,15,16]. In our previous work we presented a three dimensional finite element analysis of meniscal deformation and stress distribution in the human knee joint [17]. We have shown that changes of meniscal geometry could restrict extrusion of the torn lateral meniscus. Our analysis suggested that increase of slope angle defining the meniscus cross-section geometry could increase the force acting in radial direction which contributes to extrusion motion. Because the phenomenon described in this paper was not validated in clinical test we decided to confirm these findings in radiological trial.

## Materials and Methods

### Study participants

The experimental protocol and the study were approved by the ethics committee of the Medical University of Gdansk. All volunteers gave written informed consent to participate in the study.

Between October 2014 and September 2015 we performed MRI examination in 77 patients (34 women– 44.16%, and 43 men– 55.84%) aged 18–49 years (mean: 34.99 ± 7.98), who were complaining of knee joint disorder.

The analysis only included young (aged 20–50 years), healthy patients, with proper joint alignment, and without previous injury or diagnosed knee joint disease, in whom MRI examination failed to reveal any pathology.

Subjects less than 20 years and over 50 years old, overweight (BMI>25 kg/m^2^), with history of knee injury or disease (e.g. avascular necrosis, osteoarthritis, inflammatory disorders of the knee joint), as well as with postural abnormalities (valgus or varus deformity) were excluded from the study.

### MRI protocol

All knees were examined on a 3 T MRI scanner (Achieva TX, Philips Medical System, Eindhoven, The Netherlands) using a 16- channel extremity coil in the axial, sagittal and coronal planes with following parameters:

The spin-echo acquisition images were acquired using the following parameters: T1 coronal plane (slice thickness: 3.5 mm, FOV: 180x152x88 mm, inter-slice gap: 0.5 mm, TE: 20 ms, TR: 662 ms, matrix: 432x296) and STIR coronal plane (slice thickness: 3 mm, inter- slice gap: 1 mm, FOV: 180x151x87 mm, TE30 ms, TR/TI: 3636/190 ms, matrix size: 512x512 pixels).

The spin-echo proton density weighted acquisition images with following parameters: axial (slice thickness: 3.5 mm, FOV: 190x157x96 mm, inter-slice gap: 0.5 mm, TE: 30 ms, TR: 2888 ms, matrix: 456x259) and sagittal (slice thickness: 3.5 mm, FOV: 170x170x92 mm, inter-slice gap: 0.5 mm, TE: 30 ms, TR: 1411 ms, matrix size: 512x512 pixels).

T2-weighted images were also acquired using the following parameters for axial images: slice thickness: 3.5 mm, FOV: 190x157x96 mm, inter-slice gap: 0.5 mm, TE: 12 ms, TR: 435 ms, matrix size: 512x512 pixels).

For this study were analyzed only coronal (T1 and STIR) scans in the level of the intercondylar eminence because they provide optimal delineation of the meniscus body. Other sequences were used for an overall assessment of the knee.

### MRI interpretations

The process of reviewing the 77 MR images was performed.

The extent of lateral meniscus extrusion was evaluated using a professional workstation (MR Workspace) with professional software (Extended MR workspace 2.6.3.5, 2013).

On the coronal image at the mid-point of the lateral femoral condyle, extrusion of the lateral meniscus from the margin of the lateral tibial plateau was evaluated.

**Fig 1:**
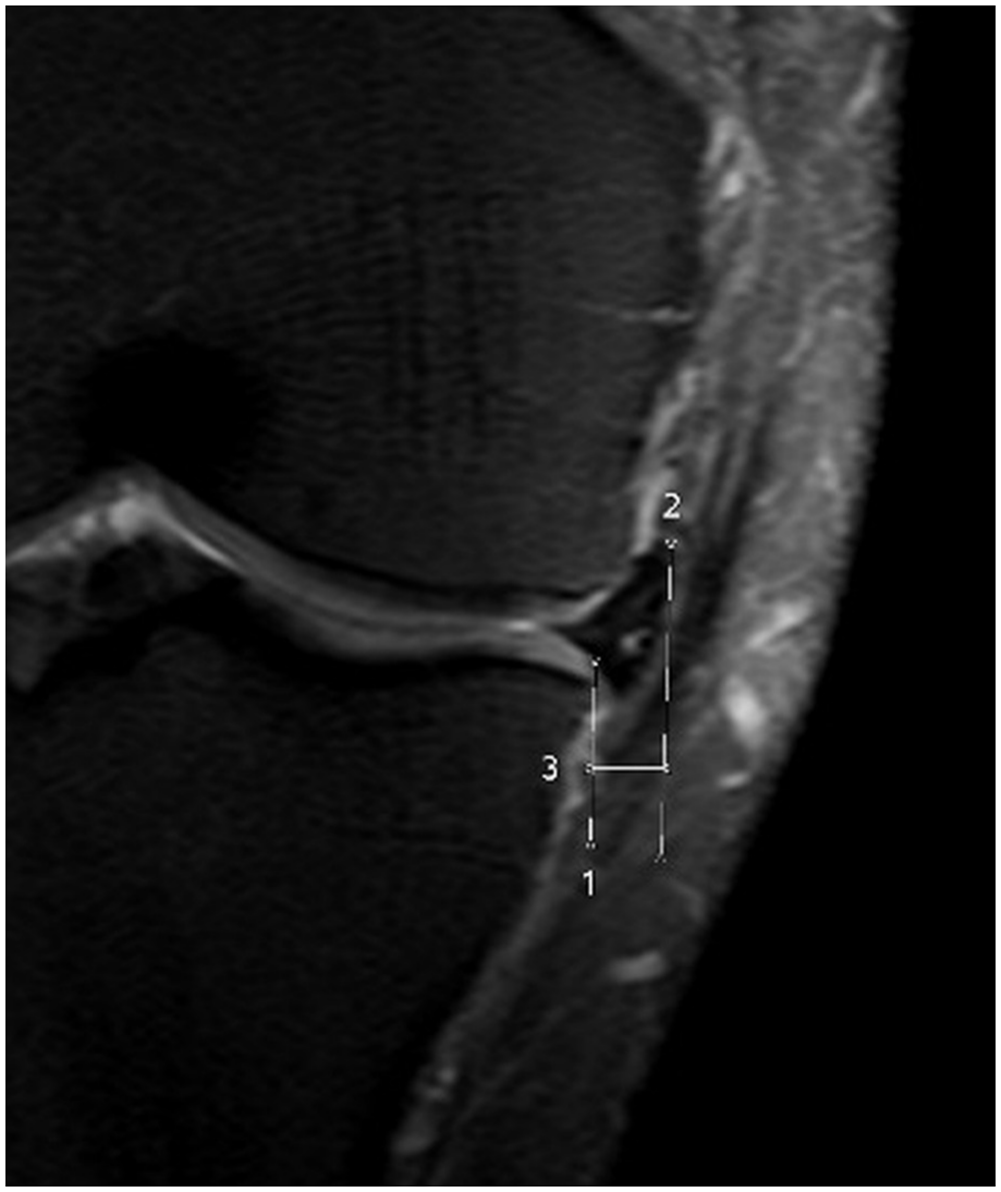
Meniscal extrusion is defined as the greatest distance (line No 3) from the most peripheral aspect of the meniscus (line No 2) to the border of the tibial plateau (line No 1).

The measurement was performed by first drawing a vertical line intersecting the peripheral margin of the lateral tibial plateau at the point of transition from horizontal to vertical; the length of another line extending from the first line to the outer margin of the meniscus was defined as meniscal extrusion (Fig 1).

Patients were divided into two groups: in the absence of extrusion of meniscus (N: 48), and with the extrusion (N: 29).

**Fig 2:**
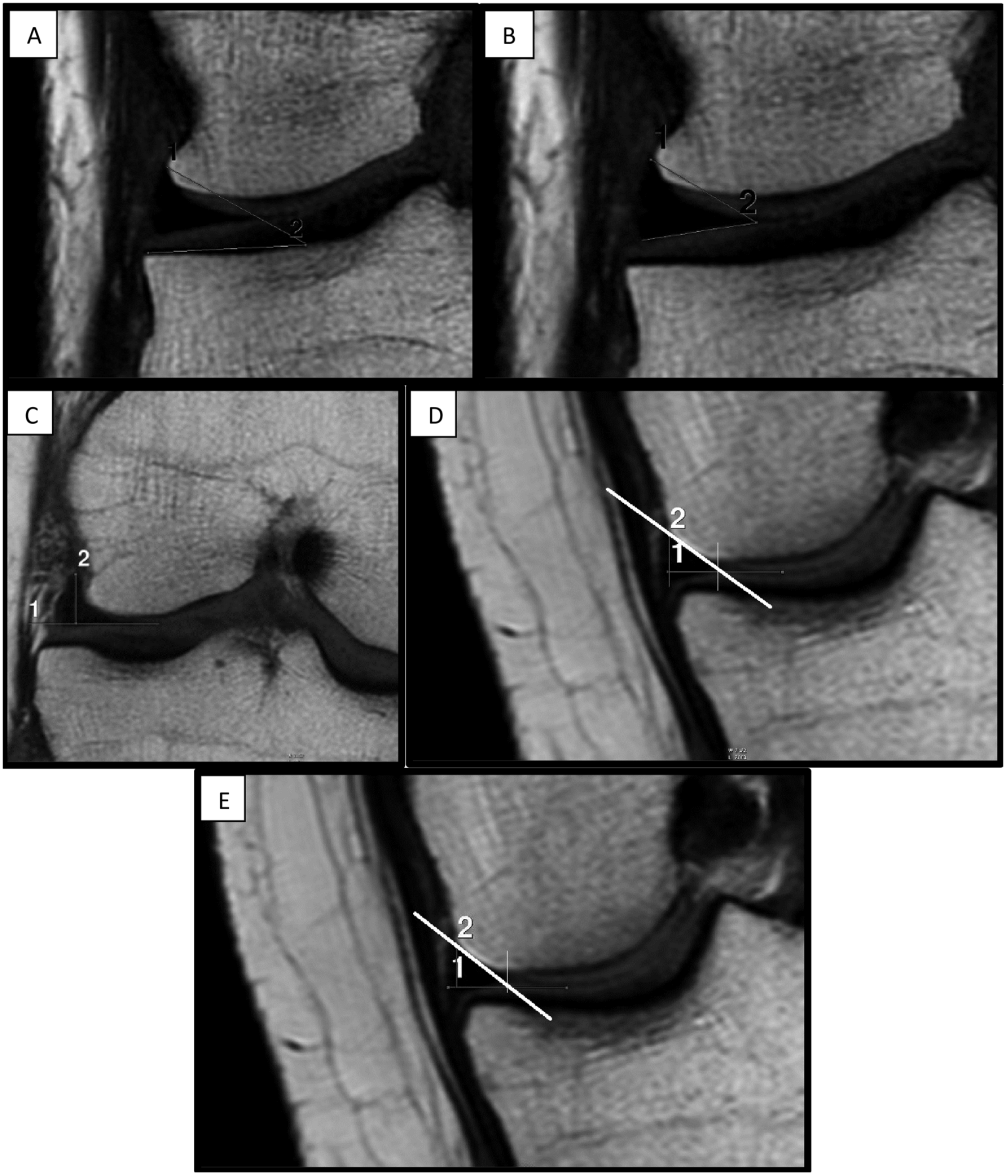
A-E. The central slice of a coronal MRI imaging of the knee focused on lateral compartment: A) meniscus-bone angle, B) meniscus-cartilage angle, C) meniscus-cartilage height, D) slope A, E) slope angle.

The following parameters were used in the analysis of the shape of the lateral meniscus (Fig 2A–2E):

1. MBA (meniscus-bone angle) (expressed in degrees)—angle between tibial plateau and superior margin of the meniscus (Fig 2A),
2. MCA (meniscus-cartilage angle) (expressed in degrees)—angle between superior and inferior margin (maximum meniscal height) of the meniscus (Fig 2B),
3. MCH (meniscus-cartilage height) (expressed in millimeters)–distance between the top of the shaft and a line drawn along the superior margin of tibial cartilage (Fig 2C),
4. slope A (expressed in degrees)–equal to the arctangent of the quotient of maximum meniscal height (2) to its width (1), determined according to the following equation: slope A = arctan (2/1) (Fig 2D),
5. slope angle (expressed in degrees)–equal to the arctangent of the quotient of MCH (2) to meniscal width (1), determined according to the following equation: slope angle = arctan (2/1) (Fig 2E).

All MRI studies were assessed retrospectively by two radiologists (AS, MS) with, respectively, ten years and four years of experience in assessment of musculoskeletal system examinations. The raters did not know the purpose of the study; they were blind to study participant characteristics and study objectives. Two radiologists examined each MRI study and performed measurements by reaching a consensus. All data and related metadata underlying the findings reported in this article is available as Supporting Information (S1 Table).

### Statistical Analysis

All data were presented as a mean ± standard deviation. Slope A and slope angle values were calculated as arctangent function of ratio respectively height A or height MCH to the length of the meniscus. This provided slopes in degrees. Differences between mean values in groups with (E) and without (NE) extrusion were examined by Welch’s t test for independent samples or Mann-Whitney U test if necessary. Normality assumption was verified using the W Shapiro-Wilk test. Linear interdependence between quantitative variables was assessed by Pearson's correlation coefficient values.

To find out which of the presented variables were associated with the risk of extrusion, the logistic regression was used. After identifying the risk factors receiver operating characteristic (ROC) analysis was used to determine the power of the predictor and cut-off point designated by Youden’s index.

Additionally, Kaplan-Meier's survival function was performed where survival time was set as given risk factor and censoring indicator as the presence of extrusion (dichotomous variable). The median of this survival function gave information when (for which value) the probability of extrusion presence is equal to its non-presence.

The level of significance was set at α = 0.05. All calculated p-values were for two-tailed tests. All raw data were analyzed using statistical software Statistica 12.5 (StatSoft, Inc. 2014).

## Results

The analysis revealed statistically significant differences between mean values of MCH (p = 0.010), MCA (p = 0,046), MBA (p = 0.003), slope A (0.022) and slope angle (p<0.001) in groups with (E) and without (NE) extrusion (Table 1). None of BMI and Age differed significantly.

**Table 1:** Test results for difference between the groups NE and E.

Variable	Test results Number of observations: NE– 48, E– 29			
	Mean ± SD		t/z	p
	NE	E		
Age	34.79 ± 7.49	35.31 ± 8.86	-0.442	0.659
BMI	23.09 ± 3.03	23.34 ± 3.59	-0.037	0.971
MCH	4.96 ± 0.89	5.75 ± 1.39	-2.712	0.010
Slope A	36.22 ± 6.85	39.29 ± 4.67	-2.337	0.022
Slope angle	27.24± 6.13	32.63± 5.98	-3.798	<0.001
MCA (degree)	44.77 ± 10.14	50.03 ± 11.43	-2.042	0.046
MBA (degree)	31.17 ± 7.31	37.83 ± 10.05	-3.106	0.003

Groups with (E) and without (NE) extrusion; SD- standard deviation, BMI—body mass index, MCH—meniscus-cartilage height; MCA—meniscus-cartilage angle.

Strong positive correlation (Table 2) existed between the pairs slope A and MCA (r = 0.815, p<0.001), MCA and MBA (r = 0.789, p<0.001), MBA and slope angle (r = 0.767, p<0.001), slope angle and MCA (r = 0.698, p<0.001).

**Table 2:** Corelation Coeficients matrix for MCH, MCA, MBA, Slope A and Slope angle.

Variable	Pearson’s Corelation Coeficients.				
	MCH	MCA	MBA	Slope A	Slope angle
MCH	1.000	0.175	0.397	0.229	0.647
MCA	0.175	1.000	0.789	0.815	0.698
MBA	0.397	0.789	1.000	0.650	0.767
Slope A	0.229	0.815	0.650	1.000	0.781
Slope angle	0.647	0.698	0.767	0.781	1.000

MCH—meniscus-cartilage height; MCA—meniscus-cartilage angle; MBA—meniscus-bone angle.

Statistically significant (p<0.001) logit model (Table 3) suggests that mainly MBA (p = 0.024) and little MCH (p = 0.065) determine the risk of extrusion. The growth of MBA about 1 degree a risk of extrusion increases 1.078 times and with the growth of 1 mm of MCH the risk grows 1.593 times. The total predictability of this model is 67.53%.

**Table 3:** Logistic regression results—model 1.

N = 77	Logistic regression (logit) N of 0's: 29 1's: 48 Chi^2(2) = 14,156 p = 0,00084		
	Const. B_0_	MCH	MBA
Estimate	-5.546	0.466	0.075
Standard Error	1.599	0.252	0.033
Wald's Chi-square	12.031	3.414	5.085
p-value	0.001	0.065	0.024
Odds ratio		1.593	1.078

Next statistically significant (p<0.001) logit model (Table 4) takes into account only slope angle (p = 0.001). Rising the slope angle with 1 degree increases the risk of extrusion about 1.157 times. The total predictability of this model is 68.83%.

**Table 4:** Logistic regression results—model 2.

N = 77	Logistic regression (logit) N of 0's: 29 1's: 48 Chi^2(1) = 13,186 p = 0,00028	
	Const. B_0_	Slope angle
Estimate	-4.872	0.146
Standard Error	1.384	0.045
Wald's Chi-square	12.384	10.711
p-value	<0.001	0.001
Odds ratio		1.157

**Fig 3:**
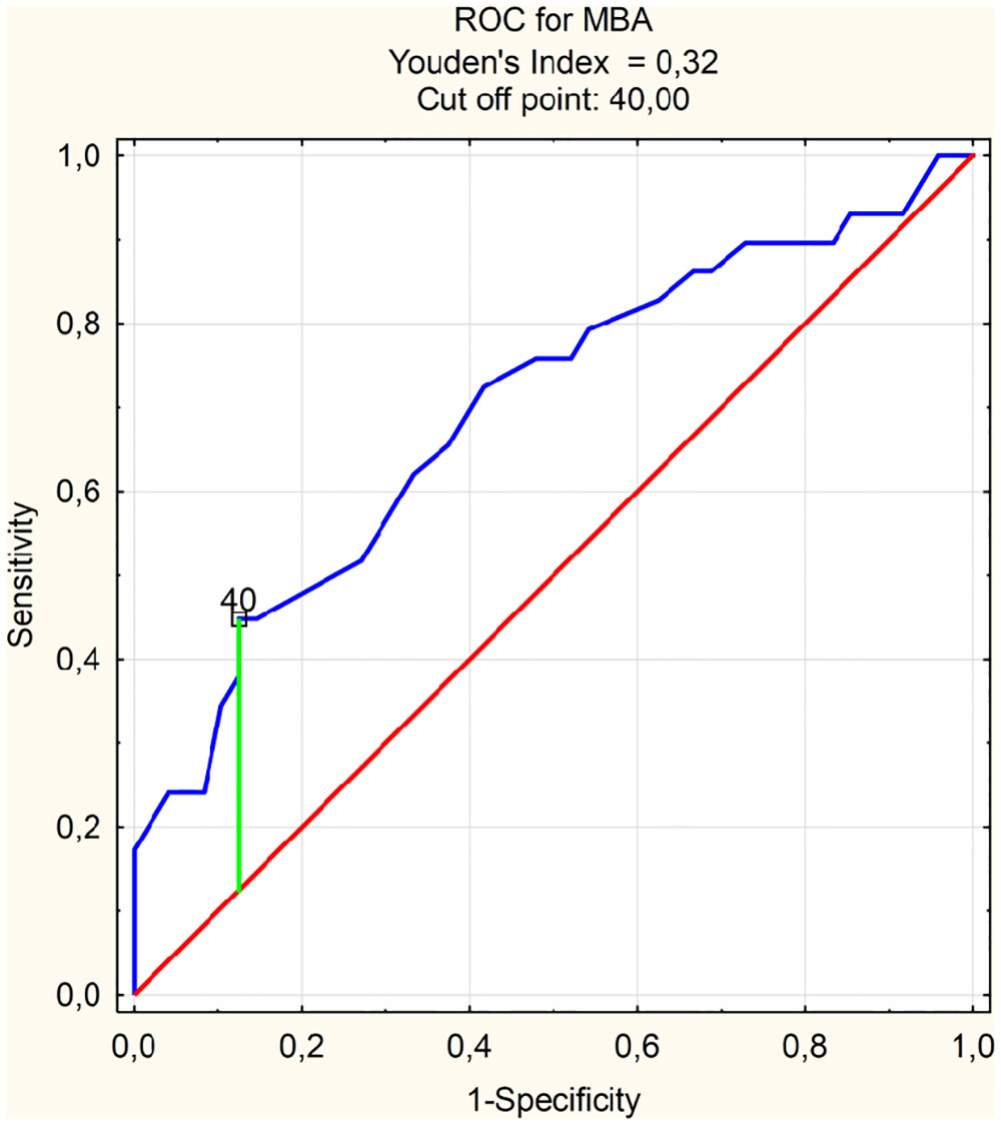
ROC and cut off point for MBA. ROC—receiver operating characteristic.

**Fig 4:**
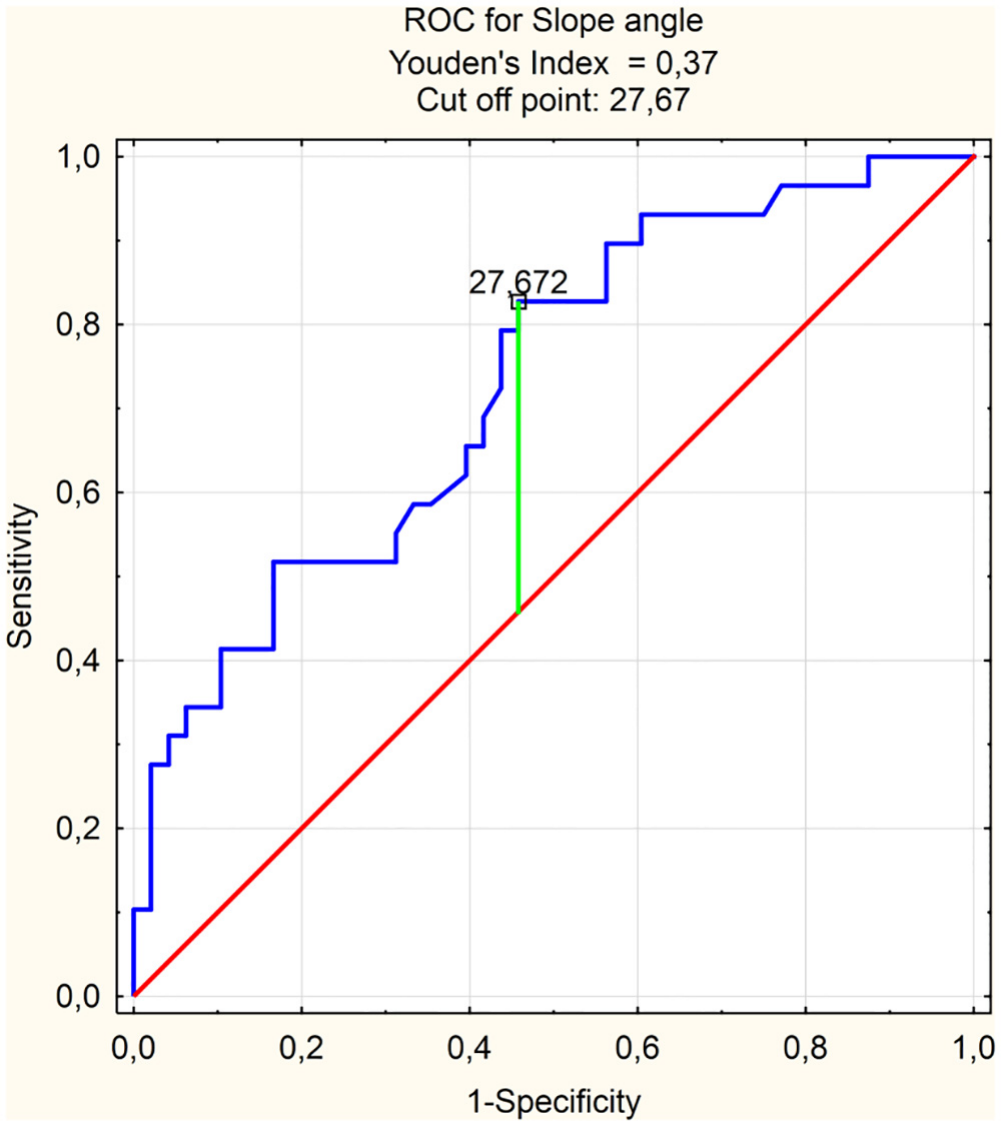
ROC and cut off point for Slope angle. ROC—receiver operating characteristic.

The ROC analysis indicated that previously specified by logistic regression extrusion risk factors like MBA (AUC = 0.697) and slope angle (AUC = 0.729) had prediction ability at an acceptable level (Table 5). The cut-off point for MBA was 40.00 (Fig 3) while sensitivity and specificity at this level respectively 0.448 and 0.875. Similarly for slope angle cut off point 27.70 (Fig 4) marked sensitivity at 0.828 and specificity as 0.542.

**Table 5:** ROC analysis results for MBA and Slope angle.

	ROC analysis results				
	AUC	SE	z	p	Cut off point
MBA	0.697	0.063	3.101	0.002	40.0°
Slope angle	0.729	0.059	3.907	<0.001	27.7°

ROC—receiver operating characteristic; MBA—meniscus-bone angle.

**Fig 5:**
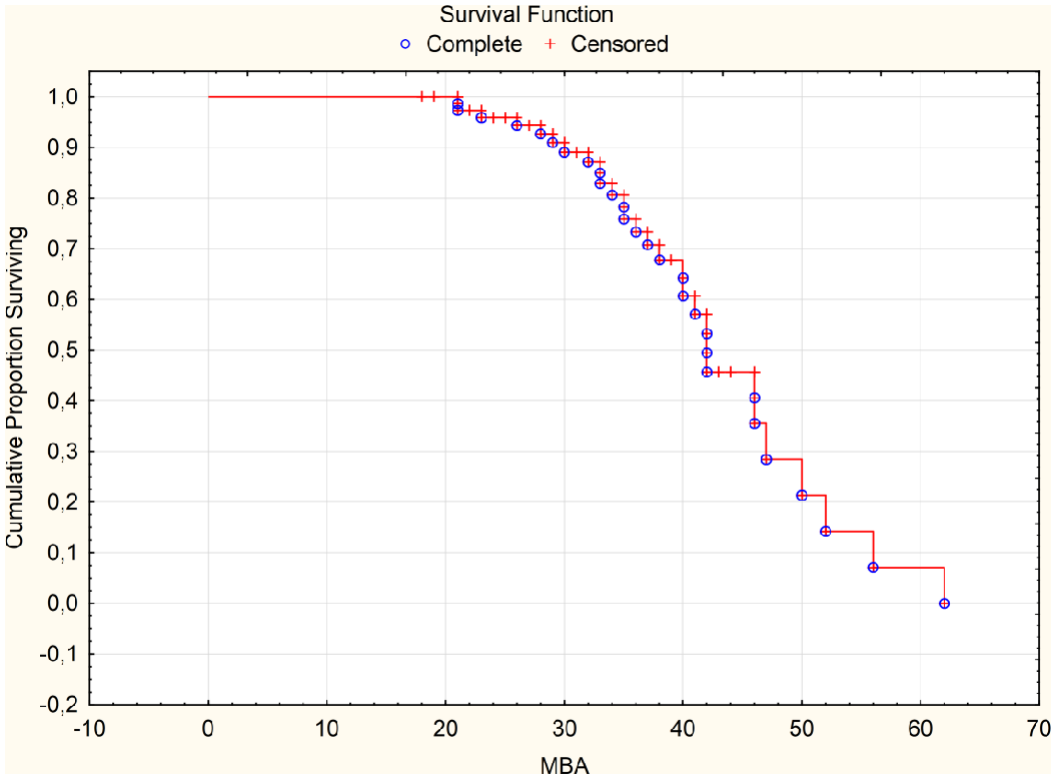
Survival Function for MBA. MBA—meniscus-bone angle.

**Fig 6:**
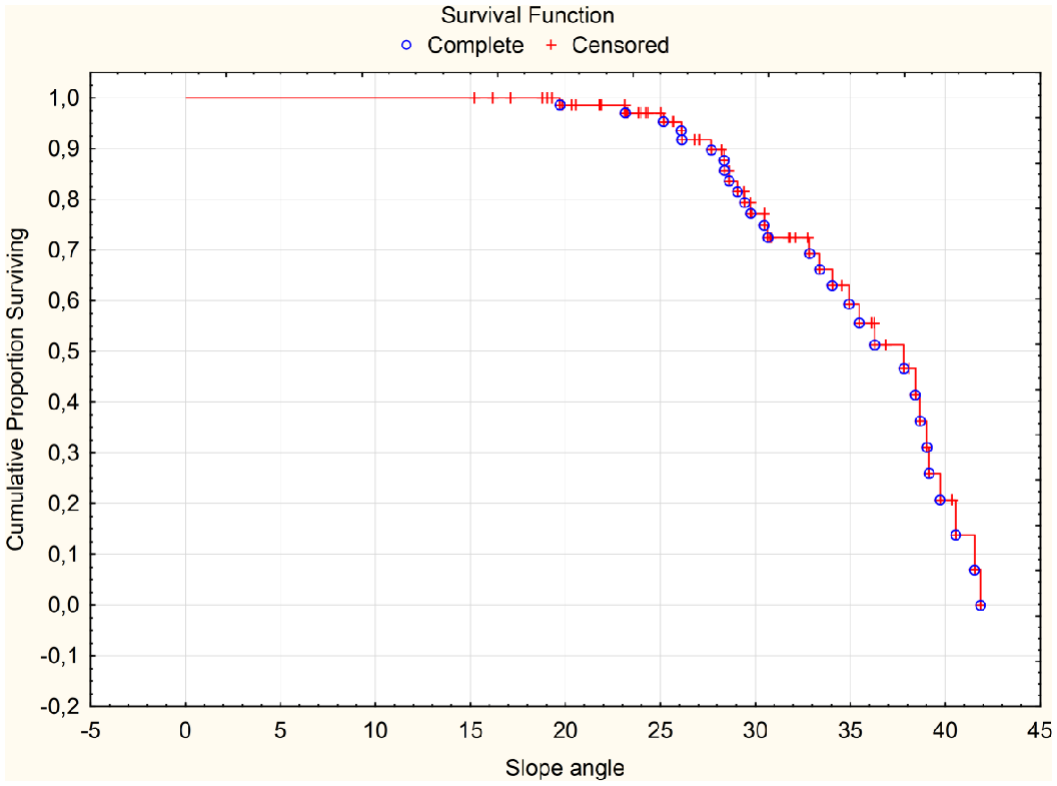
Survival Function for Slope angle.

The median values of survival functions estimated for MBA (Fig 5) and slope angle (Fig 6) were approximately respectively 420 and 370. Because the growth of MBA and slope angle increases the risk of extrusion presence then for angles greater than median values it is more likely to occur.

## Discussion

The goal of this work was to verify the results of Luczkiewicz et al. [17] regarding a relationship between the shape of the meniscus and the risk of its extrusion. In that study, based on a mathematical model of a knee joint using finite element method, a tight correlation was demonstrated between an increase of the angle of inclination of the superior meniscal surface (slope angle) and increase in forces acting in radial direction, which is responsible for extrusion in the medial part of the meniscus.

Although findings were consistent with intuitive expectations, the mathematical model did not possess physical validation. For that reason, we decided to conduct an additional clinical study. Previous studies have shown that extrusion can be associated with joint space narrowing, radial meniscal tear and varus deformity [4]. Therefore, we excluded patients with the above-mentioned pathologies from our study group.

We demonstrated a correlation between the value of slope angle, MBA and MCH and the probability of lateral meniscus extrusion, which confirmed the results of earlier studies based on mathematical analyses (finite element method) [17].

The risk factors for meniscal extrusion are quite well described in the literature [1,4,13,14,18,19,20,21]. Nevertheless, the vast majority of authors focused on the assessment of the influence of meniscal tears or change in biomechanical conditions of the knee joint on the risk of development of this pathology [4,13,14,18].

Nakamura et al. evaluated an association between the shape of tibial spurs and lateral meniscal displacement. They observed a relationship between tibial morphology and meniscal luxation only in the medial compartment [13].

Sturnick et al. [18] demonstrated a relationship between the geometry of the posterior meniscal horn and anterior cruciate ligament (ACL) injury. They revealed that increased slope of the articular cartilage and reduced height of the posterior horn of the meniscus were associated with elevated risk of non-contact ACL injury. The study findings suggest a direct link between the geometry of the meniscus with articular cartilage and knee laxity. While previous reports focused on the analyses of posterior meniscal horn geometry, those analyses concern geometry of the central part of the meniscus.

In our study, we identified a new parameter in the radiological assessment of the knee joint, such as the slope angle. It is most useful in predicting the risk of lateral meniscus extrusion. According to the ROC analysis the cut-off point for this parameter is 27 degrees (Fig 4), while the risk of extrusion increases significantly over the value of 37 degrees (Fig 6). Taking into consideration a dynamic character of this phenomenon of meniscal luxation, measurements of the above parameter can find practical applications in the assessment of the risk of instability, which correlates clinically with patients’ pain perception in degenerative meniscal lesions [4,14,9,22]. Degenerative meniscal tears are not directly associated with symptoms [7] in spite of meniscal tears with extrusion which are strongly associated with knee pain [4]. The parameter evaluated by our team could be used as an additional risk factor for estimation of extrusion in patients with degenerative damage to the meniscus. Moreover, this parameter appears useful in selecting the best-suited allograft for meniscal transplantation. Meniscal graft extrusion is a common problem in the meniscus-transplanted knee [23]. One hypothesized cause of this phenomenon is over-sizing of the allograft [24]. Nowadays, method used for the estimation of meniscal graft size relies on measurements of width and length of meniscal allograft [25]. We believe that the results of our work allow for the introduction of the additional parameter that allows a better fit of the allograft to the shape of the uninjured knee meniscus.

MBA was another analyzed parameter. ROC curve determines the cut-off point at 40 degrees (Fig 3). Above 42 degrees the risk of lateral meniscus extrusion grows significantly (Fig 5).

Meniscal- cartilage height (MCH) was an important factor (although statistically less significant than the others) influencing the probability of extrusion. We showed that an increase in this parameter by 1 mm raises the risk of extrusion by 1.593 (Table 3).

Sturnick et al. demonstrated a tight correlation between the risk of ACL injury and meniscal height (MCH) as well as middle-cartilage slope (MCS) value in the lateral knee joint compartment [18]. These results are concordant with our study. Even though our analyses did not concern assessment of meniscal horns (we studied the core) or the risk factors for ACL injury, we confirmed (after Sturnick) the importance of a parameter describing the probability of knee joint pathology in the lateral compartment, such as meniscal- cartilage height (MCH).

The above analyses seem to confirm that lateral meniscus protrusion is a multifactorial process, involving not only its shape, but also structure and position of the tibia. Also, one should not forget about an important risk factor for meniscal dislocation, such as joint injury.

We believe that study group selection may be one of the limitations of our study. In our analysis we only included young patients with normal BMI, and no medical history of knee injury or other disorders. Meniscal extrusion, although indisputably harmful from the biomechanical point of view, is often asymptomatic in the healthy population. In our study meniscal extrusion occurred in approximately 37%. According to available literature, this result does not differ from the average for the population of young, healthy people [2].

Moreover, statistical analyses of our material did not show a clear relationship between the risk of extrusion and borderline values of studied parameters. Therefore, we used advanced statistical methods to verify the thesis put forward at the beginning of this publication. The survival functions are widely used for description “time-to-event” variables. However “time” is nothing but continuous variable and that is why all techniques and tools of survival analysis (especially Kaplan-Meier estimator) are also adapted for measuring other continuous variables. It is very popular among engineers when testing the strength of materials. In material engineering for example survival function for ceramic materials “time-to-event” variable is expressed by “strength in MPa units” [26] or in another example survival function of shock-absorber lifetime “time-to-event” variable is presented by “kilometers of vehicle use” [27]. Application of survival function is based on a similar reasoning as in material engineering. Increasing a factor (in our case, "slope") eventually leads to the occurrence of the event ("extrusion").

Nevertheless, the results of our studies gave similar conclusions we regard that problem of determining the risk factors of meniscal extrusion is still open.

## Conclusions

The results of this study are further confirmed by the outcome of the mathematical model, which indicates a correlation between the shape of the meniscus and the risk of its displacement. According to our analysis the rise of the slope angle, MBA and MCH defining the meniscus cross-section geometry, could increase the risk of meniscal extrusion.

We believe that the above-mentioned parameters may be useful, not only in the assessment of the risk of meniscal extrusion, particularly with coexisting degenerative changes but also in the better selection of the allograft for meniscal transplantation.

## Supporting Information

S1 Table (XLS)

## References

[1] Hwang SH, Jung KA, Lee WJ, Yang KH, Lee DW, Carter A, et al Morphological changes of the lateral meniscus in end stage lateral compartment osteoarthritis of the knee. Osteoarthritis and cartilage. 2012;20: 110–116. 10.1016/j.joca.2011.11.005 22133800

[2] Rennie WJ, Finlay DB. Meniscal extrusion in young athletes: associated knee joint abnormalities. AJR Am J Roentgenol. 2006;186: 791–794. 16498108 10.2214/AJR.04.1181

[3] Lee DH, Lee BS, Kim JM, Yang JS, Cha EJ, Park JH et al: Predictors of degenerative medial meniscus extrusion: radial component and knee osteoarthritis. Knee Surg Sports Traumatol Arthrosc. 2011;19: 222–229. 10.1007/s00167-010-1274-2 20890696

[4] Wenger A, Englund M, Wirth W, Hudelmaier M, Kwoh K, Eckstein F et al Relationship of 3D meniscal morphology and position with knee pain in subjects with knee osteoarthritis: a pilot study. Eur Radiol. 2012;22: 211–20. 10.1007/s00330-011-2234-z 21842432

[5] Bloecker K, Wirth W, Guermazi A Hunter DJ, Resch H, Hochreiter J et al Relationship between medial meniscal extrusion and cartilage loss in specific femorotibial subregions: Data from the osteoarthritis initiative. Arthritis Care & Research. 2015;67: 1545–1552. 25988986 10.1002/acr.22615 PMC4624520

[6] Englund M, Niu J, Guermazi A, Roemer FW, Hunter DJ, Lynch JA, et al Effect of meniscal damage on the development of frequent knee pain, aching, or stiffness. Arthritis Rheum. 2007;56: 4048–4054. 18050201 10.1002/art.23071

[7] Englund M, Guermazi A, Gale D, Hunter DJ, Aliabadi P, Clancy M, et al Incidental meniscal findings on knee MRI in middle-aged and elderly persons. N Engl J Med. 2008;359: 1108–1115. 10.1056/NEJMoa0800777 18784100 PMC2897006

[8] Costa CR, Morrison WB, Carrino JA. Medial meniscus extrusion on knee MRI: is extent associated with severity of degeneration or type of tear? Am J Roentgenol. 2004;183: 17–23. 15208101 10.2214/ajr.183.1.1830017

[9] Sharma L, Eckestein F, Song J, Guermazi A, Prasad P, Almagor O, et al Relationship of meniscal damage, meniscal extrusion and joint laxity to subsequent cartilage loss in osteoarthritic knees. Arthritis Rheum. 2008;58: 1716–1726. 10.1002/art.23462 18512777

[10] Sugita T, Kawamata T, Ohnuma M, Yoshizumi Y, Sato K. Radial displacement of the medial meniscus in varus osteoarthritis of the knee. Clin Orthop Relat Res. 2001; 387: 171–177. 11400879 10.1097/00003086-200106000-00023

[11] Gale DR, Chaisson CE, Totterman SM, Schwartz RK, Gale ME, Felson D. Meniscal subluxation: association with osteoarthritis and joint space narrowing. Osteoarthritis Cartilage. 1999;7: 526–532. 10558850 10.1053/joca.1999.0256

[12] Hunter DJ, Zhang YQ, Tu X, Lavalley M, Niu JB, Amin S, et al Change in joint space width: hyaline articular cartilage loss or alteration in meniscus? Arthritis Rheum. 2006;54: 2488–2495. 16868968 10.1002/art.22016

[13] Nakamura N, Sumen Y, Sakaridani K, Exham H, Ochi M. Relationship between the shape of tibial spurs on X-ray and meniscal changes on MRI in early osteoarthritis of knee. Magnetic Resonance Imaging 2006;24: 1143–1148. 17071336 10.1016/j.mri.2006.07.006

[14] Wenger A, Wirth W, Hudelmaier M, Noebauer-Huhmann I, Trattnig S, Bloecker K, et al Meniscus body position, size, and shape in persons with and persons without radiographic knee osteoarthritis: quantitative analyses of knee magnetic resonance images from the osteoarthritis initiative. Arthritis Rheum. 2013;65: 1804–11. 10.1002/art.37947 23529645

[15] Chan WP, Huang GS, Hsu SM, Chang YC, Ho WP. Radiographic joint space narrowing in osteoarthritis of the knee: relationship to meniscal tears and duration of pain. Skeletal Radiol. 2008;37: 917–922. 10.1007/s00256-008-0530-8 18594811 PMC2525847

[16] Raynauld JP, Martel-Pelletier J, Berthiaume MJ, Beaudoin G, Choquette D, Haraoui B. Long term evaluation of disease progression through the quantitative magnetic resonance imaging of symptomatic knee osteoarthritis patients: correlation with clinical symptoms and radiographic changes. Arthritis Res Ther. 2006;8: 21–33. 10.1186/ar1875 PMC1526551 16507119

[17] Luczkiewicz P, Daszkiewicz K, Witkowski W, Chroscielewski J, Zarzycji W. Influence of meniscus shape in the cross sectional plane on the knee contact mechanics. J Biomech. 2015;48: 1356–1363. 10.1016/j.jbiomech.2015.03.002 25892539

[18] Sturnick DR, Van Gorder R, Vacek PM, DeSarno MJ, Gardner-Morse MG, Tourville TW. Tibial Articular Cartilage and Meniscus Geometries Combine to Influence Female Risk of Anterior Cruciate Ligament Injury. J Orthop Res 2014;32: 1487–1494. 10.1002/jor.22702 25099246 PMC6886124

[19] Arno S, Walker PS, Bell CP, Krasnokutsky S, Samuels J, Abramson SB, et al: Relation between cartilage volume and meniscal contact in medial osteoarthritis of the knee. The Knee. 2012;19: 896–901. 10.1016/j.knee.2012.04.005 22560645 PMC3684170

[20] Khan N, McMahon P, Obaid H. Bony morphology of the knee and non-traumatic meniscal tears: Is there a role for meniscal impingement? Skeletal Radiol. 2014;43: 955–962. 10.1007/s00256-014-1867-9 24722655

[21] Hudek R, Fuchs B, Regenfelder, Koch PP. Is Noncontact ACL Injury Associated with the Posterior Tibial and Meniscal Slope? Clin Orthop Relat Res. 2011;469: 2377–2384. 10.1007/s11999-011-1802-5 21318628 PMC3126958

[22] Sharma L, Song J, Felson DT, Cahue S, Shamiyeh E, Dunlop DD. The role of knee alignment in disease progression and functional decline in knee osteoarthritis. JAMA 2001;286: 188–95. 11448282 10.1001/jama.286.2.188

[23] Lee BS, Kim JM, Bin SI, Kim KA, Bim SU. Patient-related risk factors for the extrusion of lateral meniscal allograft transplants. Arthroscopy. 2015;31: 699–706. 10.1016/j.arthro.2014.10.016 25530512

[24] Jang SH, Kim JG, Ha JG, Shim JC. Reducing the size of the meniscal allograft decreases the percentage of extrusion after meniscal allograft transplantation. Arthroscopy. 2011;27: 914–22. 10.1016/j.arthro.2011.02.017 21693346

[25] Pollard ME, Kang Q, Berg EE. Radiographic sizing for meniscal transplantation. Arthroscopy. 1995;11: 684–7. 8679029 10.1016/0749-8063(95)90110-8

[26] Basu B, Tiwari D, Kundu D, Prasad R. Is Weibull distribution the most appropriate statistical strength distribution for brittle materials, Ceramics International. 2009;35: 237–246.

[27] O'Connor P, Kleyner A. Practical Reliability Engineering, New York: Wiley 1985.

